# Bradykinin and adenosine receptors mediate desflurane induced postconditioning in human myocardium: role of reactive oxygen species

**DOI:** 10.1186/1471-2253-10-12

**Published:** 2010-07-29

**Authors:** Sandrine Lemoine, Clément Buléon, René Rouet, Calin Ivascau, Gérard Babatasi, Massimo Massetti, Jean-Louis Gérard, Jean-Luc Hanouz

**Affiliations:** 1 Laboratory of Experimental Anesthesiology and Cellular Physiology, IFR 146 ICORE, Université de Caen Basse Normandie, CHU Caen, Avenue de la Cote de Nacre, 14033 Caen, France; 2 Department of Anesthesiology, CHU Caen, Avenue de la Cote de Nacre, 14033 Caen, France; 3 Department of cardiac and thoracic surgery, CHU Caen, Avenue de la Cote de Nacre, 14033 Caen, France

## Abstract

**Background:**

Desflurane during early reperfusion has been shown to postcondition human myocardium, *in vitro*. We investigated the role of adenosine and bradykinin receptors, and generation of radical oxygen species in desflurane-induced postconditioning in human myocardium.

**Methods:**

We recorded isometric contraction of human right atrial trabeculae hanged in an oxygenated Tyrode's solution (34 degrees Celsius, stimulation frequency 1 Hz). After a 30-min hypoxic period, desflurane 6% was administered during the first 5 min of reoxygenation. Desflurane was administered alone or with pretreatment of *N*-mercaptopropionylglycine, a reactive oxygen species scavenger, 8-(p-Sulfophenyl)theophylline, an adenosine receptor antagonist, HOE140, a selective B2 bradykinin receptor antagonist. In separate groups, adenosine and bradykinin were administered during the first minutes of reoxygenation alone or in presence of *N*-mercaptopropionylglycine. The force of contraction of trabeculae was recorded continuously. Developed force at the end of a 60-min reoxygenation period was compared (mean ± standard deviation) between the groups by a variance analysis and post hoc test.

**Results:**

Desflurane 6% (84 ± 6% of baseline) enhanced the recovery of force after 60-min of reoxygenation as compared to control group (51 ± 8% of baseline, *P *< 0.0001). *N*-mercaptopropionylglycine (54 ± 3% of baseline), 8-(p-Sulfophenyl)theophylline (62 ± 9% of baseline), HOE140 (58 ± 6% of baseline) abolished desflurane-induced postconditioning. Adenosine (80 ± 9% of baseline) and bradykinin (83 ± 4% of baseline) induced postconditioning *(P *< 0.0001 *vs *control), *N*-mercaptopropionylglycine abolished the beneficial effects of adenosine and bradykinin (54 ± 8 and 58 ± 5% of baseline, respectively).

**Conclusions:**

*In vitro*, desflurane-induced postconditioning depends on reactive oxygen species production, activation of adenosine and bradykinin B_2 _receptors. And, the cardioprotective effect of adenosine and bradykinin administered at the beginning of reoxygenation, was mediated, at least in part, through ROS production.

## Background

Anesthetic-induced postconditioning (PostC) is a phenomenon whereby a brief exposure of the myocardium to a volatile halogenated anesthetic, at the very onset of reperfusion, markedly reduces myocardial injury following prolonged ischemia: anesthetic-induced PostC has been confirmed in several mammalian species including rat, mouse, rabbit, and human [1-8].

The mechanism of volatile anesthetic-induced decrease of reperfusion injury remains incompletely understood. Endogenous activation of opioids, bradykinin, and adenosine receptors can trigger the complex protective signalling pathway of ischemic PostC [9]. It has been shown that adenosine and bradykinin postconditioned isolated rabbit [10] and rat hearts [11], via stimulation of adenosine and B_2 _receptors. At present, the involvement of adenosine and bradykinin receptors in anesthetic-induced PostC remains unknown, whereas these receptors were shown to be involved in anesthetic-induced preconditioning [12]. On the other hand, several studies showed that volatile anesthetic triggered intracellular reactive oxygen species (ROS) production [13], and that ROS production may mediate and/or trigger the preconditioning signalling cascade. Thus, sevoflurane and desflurane-induced preconditioning were abolished by ROS scavengers [14]. However, only two studies suggested that ROS were involved in isoflurane-induced PostC in mouse myocardium *in vivo *[3], and in sevoflurane-induced PostC in isolated rat hearts [8].

The objectives of our study were to determine whether: 1) ROS generation, and adenosine and bradykinin receptor stimulation may be involved in desflurane-induced PostC, 2) adenosine and bradykinin given at the beginning of reoxygenation mimic PostC, 3) adenosine and bradykinin receptors' activation was followed by myocardial PostC via ROS production.

## Methods

After the approval of local medical ethics committee (Comité de Protection des Personnes Nord Ouest III, Caen, France) and written informed consent, right atrial appendages were obtained during cannulation for cardiopulmonary bypass from patients scheduled for routine coronary artery bypass surgery or aortic valve replacement. All patients received total intravenous anesthesia with propofol, remifentanil, and pancuronium. Patients with chronic atrial arrhythmia and with diabetes mellitus treated with insulin or oral hypoglycemic agents were excluded from the study [7,14].

### Experimental conditions

Right atrial trabeculae (one or two per appendage) were dissected and suspended vertically between an isometric force transducer (MLT0202, ADInstruments, Sydney, Australia) and a stationary stainless clip in a 200 ml organ bath filled with daily prepared Tyrode's modified solution containing (mM) 120 NaCl, 3.5 KCl, 1.1 MgCl_2_, 1.8 NaH_2_PO_4_, 25.7 NaHCO_3_, 2.0 CaCl_2_, and 5.5 glucose. The organ bath was maintained at 34°C by a thermostatic water circulator (Polystat micropros, Bioblock, Illkirch, France). The bathing solution was insufflated with carbogen (95% O_2_-5% CO_2_), resulting in a pH of 7.40 and a partial pressure of oxygen of 600 mm Hg. Isolated muscles were field-stimulated at 1 Hz by two platinum electrodes with rectangular wave pulses of 5 ms duration 20% above threshold (CMS 95107, Bionic Instrument, Paris, France).

Trabeculae were equilibrated for 60 to 90 min to allow stabilization of their optimal mechanical performance at the apex of the length-active isometric tension curve (L_max_). At the end of the stabilization period, trabeculae were randomized to experimental groups detailed below. The force developed was measured continuously, digitized at a sampling frequency of 400 Hz, and stored on a Writable Compact Disc for analysis (MacLab, AD Instrument, Sydney, Australia).

At the end of each experiment, the length and the weight of the muscle were measured. The muscle cross-sectional area was calculated from its weight and length assuming a cylindrical shape and a density of 1. To avoid core hypoxia, trabeculae included in the study should have a cross-sectional area less than 1.0 mm^2^, a force of contraction normalized per cross-sectionnal area (FoC) > 5.0 mN/mm^2 ^and a ratio of resting force/total force less than 0.45.

### Experimental protocol

In all groups, hypoxia-reoxygenation was performed by replacing 95% O_2_-5% CO_2 _with 95% N_2_-5% CO_2 _in the buffer for 30-min, followed by a 60-min oxygenated recovery period.

**Figure 1:**
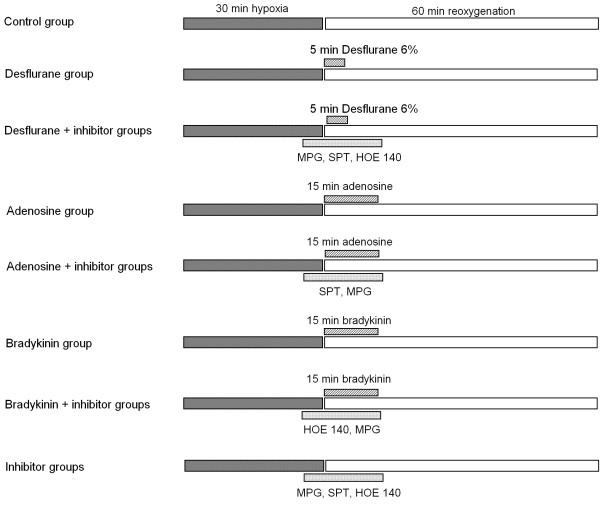
**Schematic diagram depicting the experimental protocol**. In the Desflurane + inhibitor groups and Inhibitor groups, MPG was administered at 150 μM, SPT was administered at 100 μM, and HOE 140 was administered at 20 nM. In adenosine and adenosine + inhibitor groups, adenosine was administered at 100 μM. In bradykinin and bradykinin + inhibitor groups, bradykinin was administered at 1 μM. MPG: N-mercaptopropionylglycine; SPT: 8-(p-Sulfophenyl) théophylline.

In the Control group (Control; n = 8) muscles were exposed to the hypoxia-reoxygenation protocol alone. In the desflurane treatment groups, desflurane was delivered to the organ bath by the gas flow passing through a specific calibrated vaporizer. Desflurane concentration in the carrier gas phase was measured with an infrared calibrated analyzer (Capnomac, Datex, Helsinki, Finland). Desflurane was administered at 6% (Desflurane 6%; n = 6) during the first 5 min of reoxygenation (fig. 1).

Mechanisms involved in desflurane-induced PostC were studied in presence of desflurane 6%, because we have previously shown that 6% was effective to induce PostC in human myocardium, *in vitro*.^7 ^In separate groups exposed to 6% desflurane during the first 5-min of reoxygenation in the presence of 150 μM N-mercaptopropionylglycine, a ROS scavenger (Des + MPG; n = 6), 100 μM 8-(p-Sulfophenyl)theophylline, an adenosine receptor antagonist (Des + SPT; n = 6), 20 nM HOE140, a selective B_2 _bradykinin receptor antagonist (Des + HOE; n = 6). Pharmacological agents were administered 5 min before, throughout, and 10 min after desflurane administration (fig. 1).

The effect of MPG, SPT, and HOE140 alone was studied in separate groups exposed to 150 μM N-mercaptopropionylglycine (MPG; n = 6), 100 μM 8-(p-Sulfophenyl)theophylline (SPT; n = 6), 20 nM HOE140 (HOE; n = 6), 5 min before and during the first 15 min of reoxygenation (fig. 1).

### Sequence of activation of adenosine and bradykinin receptors and ROS generation

In separate experimental groups, trabeculae were randomly assigned to receive 1) 100 μM adenosine alone, in the 15 first min of reoxygenation (adenosine (ADO) group; n = 6), or 2) co-superfused with pre-treatment (5 min before and 15 min after reoxygenation) with 100 μM SPT (ADO + SPT; n = 6), or 3) with 150 μM MPG (ADO + MPG; n = 6) (fig. 1).

In 3 other separate experimental groups, trabeculae were randomly assigned to receive: 1) 1 μM bradykinin alone, in the 15 first min of reoxygenation (BK group; n = 6), or 2) co-superfused with pre-treatment (5 min before and 15 min after reoxygenation) with 20 nM HOE140 (BK + HOE; n = 6), or 3) with 150 μM MPG (BK + MPG; n = 6) (fig. 1).

The concentrations of inhibitors MPG [14], SPT [10], HOE 140 [11], and activators adenosine [10], bradykinin [15] used here have been validated in previous experimental studies, *in vitro*.

### Chemicals

MPG and adenosine were purchased from Calbiochem (VWR International, Fontenay sous Bois, France) and bradykinin, HOE 140, SPT were obtained from Sigma Aldrich (Saint Quentin Fallavier, France). Desflurane was purchased from GlaxoWellcome (Marly-le-Roi, France).

### Statistical Analysis

The endpoint of the study was the recovery of FoC at 60 min of reoxygenation (FoC_60_, expressed as percent of baseline). All values were compared by ANOVA with application of a *post hoc *Bonferroni test. Power analysis calculated a group size of n = 5 to detect a difference of 40% in FoC (Control and inhibitors group: FoC_60 _= 50 ± 9% of baseline, and Desflurane 6% group: FoC_60_= 90 ± 9% of baseline) with a power of 0.8 at alpha-level of 0.05. The number of experiments per group was calculated based on an one-way analysis of variance (ANOVA) with 4 Control and inhibitors groups and 1 desflurane 6% group. Data are expressed as mean ± SD. Baseline values of main mechanical parameters, age, preoperative left ventricular ejection fraction, and FoC_60 _were compared by univariate analysis of variance with group factor as the independent variable. If the *P *value was less than 0.05, a Bonferroni *post hoc *analysis was performed. Within-group data were analyzed over time using two way analysis of variance for repeated measures and Bonferroni *post hoc *analysis with group factor and time (baseline, hypoxia 5, 10, 20, 30 min, and reoxygenation 5, 10, 20, 30, 40, 50, and 60 min) as independent variables.

## Results

There were no differences between groups for patients' demographic data, preoperative treatments, and left ventricular ejection fraction (Table 1). Eighty six human right atrial trabeculae were studied. There were no differences in baseline values for L_max_, cross-sectionnal area, ratio of resting force to total force, and FoC between groups (Table 2).

**Table 1:** Patients demographic data, preoperative drug treatments, and preoperative left ventricular ejection fraction

Groups and heart disease	Age (Yr)	Preoperative drug treatments	LVEF(%)
Control AVR (n = 2); CABG (n = 6)	66 ± 7	ACE (3), βAB (5), BZD (1), CA (0), COR (0), FUR (1), MOL (0), STA (5), NT (0)	73 ± 11
Desflurane AVR (n = 1); CABG (n = 5)	67 ± 9	ACE (2), βAB (5), BZD (0), CA (1), COR (0), FUR (0), MOL (1), STA (4), NT (0)	64 ± 19
Des + MPG AVR (n = 3); CABG (n = 3)	64 ± 12	ACE (2), βAB (3), BZD (1), CA (1), COR (0), FUR (0), MOL (0), STA (1), NT (0)	71 ± 21
Des + SPT AVR (n = 4); CABG (n = 2)	66 ± 21	ACE (3), βAB (3), BZD (2), CA (0), COR (0), FUR (0), MOL (1), STA (1), NT (0)	67 ± 10
Des + HOE AVR (n = 2); CABG (n = 4)	69 ± 8	ACE (4), βAB (5), BZD (2), CA (0), COR (0), FUR (0), MOL (3), STA (4), NT (2)	59 ± 2
ADO AVR (n = 1); CABG (n = 5)	70 ± 8	ACE (2), βAB (2), BZD (0), CA (1), COR (0), FUR (0), MOL (0), STA (4), NT (0)	65 ± 10
ADO + SPT AVR (n = 3); CABG (n = 3)	55 ± 11	ACE (4), βAB (4), BZD (1), CA (2), COR (0), FUR (1), MOL (0), STA (2), NT (0)	60 ± 12
ADO + MPG AVR (n = 2); CABG (n = 4)	73 ± 9	ACE (3), βAB (2), BZD (3), CA (0), COR (0), FUR (0), MOL (0), STA (3), NT (0)	73 ± 6
BK AVR (n = 5); CABG (n = 1)	67 ± 11	ACE (3), βAB (3), BZD (2), CA (2), COR (0), FUR (0), MOL (0), STA (6), NT (0)	59 ± 15
BK + HOE AVR (n = 4); CABG (n = 2)	60 ± 15	ACE (4), βAB (4), BZD (1), CA (2), COR (0), FUR (2), MOL (0), STA (3), NT (1)	65 ± 16
BK + MPG AVR (n = 3); CABG (n = 3)	66 ± 17	ACE (2), βAB (1), BZD (2), CA (0), COR (0), FUR (1), MOL (0), STA (3), NT (0)	61 ± 9
MPG AVR (n = 5); CABG (n = 1)	71 ± 2	ACE (4), βAB (2), BZD (2), CA (0), COR (0), FUR (0), MOL (0), STA (5), NT (0)	71 ± 9
SPT AVR (n = 5); CABG (n = 1)	68 ± 15	ACE (2), βAB (2), BZD (0), CA (0), COR (0), FUR (2), MOL (0), STA (2), TNT (0)	74 ± 8
HOE AVR (n = 0); CABG (n = 6)	66 ± 10	ACE (5), βAB (4), BZD (2), CA (2) COR (0), FUR (0), MOL (2), STA (4), NT (3)	70 ± 9

The number in brackets after heart disease and drug abbreviation indicate the number of patients. Age and LVEF are expressed as mean ± SD.

ACE: angiotensin-converting enzyme inhibitors; ADO: adenosine; AVR: aortic valve replacement; βAB: β-adrenergic blocking drugs; BK: bradykinin; BZD: benzodiazepine; CA: calcium channel antagonists; CABG: coronary artery bypass graft; COR: amiodarone; Des: desflurane; FUR: furosemide; HOE: HOE 140; LVEF: preoperative left ventricular ejection fraction; MOL: molsidomine; MPG: N-mercaptopropionylglycine; NT: nitroglycerin; SPT: 8-(p-Sulfophenyl) théophylline; STA: statins.

Data are mean ± SD.

**Table 2:** Control values of main mechanical parameters of human right atrial trabeculae.

Experimental Groups	L_max _(mm)	CSA (mm^2^)	FoC (mN.mm^-2^)	RF/TF
Control (n = 8)	6.8 ± 0.6	0.49 ± 0.10	24 ± 4	0.33 ± 0.03
Desflurane (n = 6)	6.5 ± 0.7	0.56 ± 0.07	25 ± 3	0.30 ± 0.04
Des + MPG (n = 6)	7.5 ± 0.6	0.56 ± 0.03	23 ± 5	0.27 ± 0.04
Des + SPT (n = 6)	6.0 ± 0.6	0.54 ± 0.06	23 ± 3	0.28 ± 0.02
Des + HOE (n = 6)	5.8 ± 0.4	0.46 ± 0.04	25 ± 3	0.34 ± 0.04
ADO (n = 6)	7.7 ± 0.8	0.59 ± 0.07	27 ± 2	0.28 ± 0.03
ADO + SPT (n = 6)	6.1 ± 0.4	0.60 ± 0.05	25 ± 3	0.24 ± 0.02
ADO + MPG (n = 6)	6.8 ± 0.9	0.44 ± 0.09	22 ± 4	0.40 ± 0.04
BK (n = 6)	5.8 ± 0.5	0.39 ± 0.06	28 ± 4	0.31 ± 0.04
BK + HOE (n = 6)	5.1 ± 0.4	0.54 ± 0.07	22 ± 2	0.29 ± 0.05
BK + MPG (n = 6)	6.0 ± 0.4	0.40 ± 0.04	23 ± 2	0.31 ± 0.03
MPG (n = 6)	6.3 ± 0.7	0.47 ± 0.11	30 ± 1	0.33 ± 0.06
SPT (n = 6)	6.6 ± 0.5	0.54 ± 0.06	26 ± 3	0.29 ± 0.03
HOE (n = 6)	5.3 ± 0.53	0.40 ± 0.05	26 ± 4	0.34 ± 0.04

L_max_: maximal length at the apex of the length-active force curve; CSA: cross sectionnal area; FoC: Force of contraction normalized per cross sectionnal area; RF/TF: ratio of resting force on total force. Data are mean ± SD.

ADO: adenosine, BK: bradykinin, Des: desflurane, HOE: HOE 140, MPG: N-mercaptopropionylglycine, SPT: 8-(p-Sulfophenyl) théophylline.

### Effects of desflurane on hypoxia reoxygenation on human atrial trabeculae

**Figure 2:**
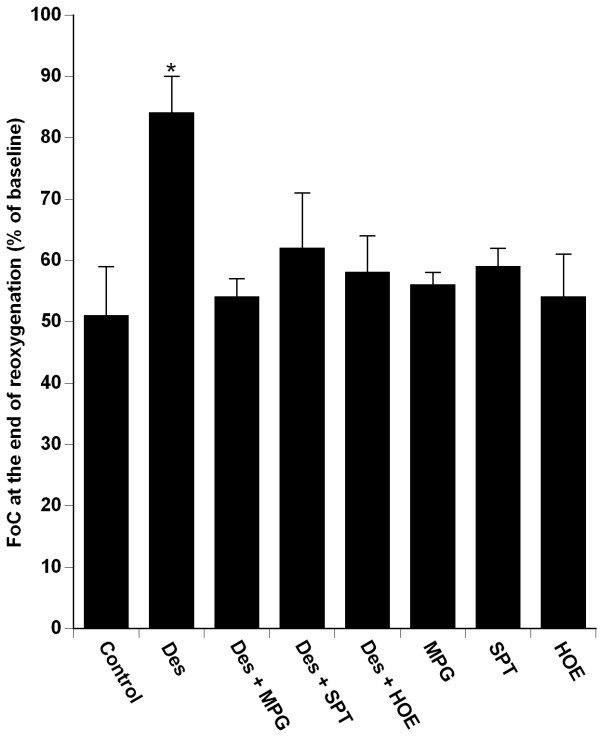
**Recovery of force of contraction of isolated human right atrial trabeculae at the end of the 60-min reoxygenation period after the 30-min hypoxic challenge in groups exposed to Desflurane 6% (Des) alone or in the presence of MPG, SPT, HOE**. Data are mean ± SD. *P < 0.0001 vs. control, Des + MPG, Des + SPT, Des + HOE, MPG, SPT, HOE. Des: desflurane; HOE: HOE 140; MPG: N-mercaptopropionylglycine; SPT: 8-(p-Sulfophenyl) théophylline.

In the Control group, reoxygenation resulted in a partial recovery of FoC (FoC_60_: 51 ± 8% of baseline). Desflurane increased the FoC_60 _as compared to Control group (FoC_60_: 84 ± 6% of baseline *vs*. Control; *P *< 0.0001) (fig. 2).

### Effects of N-mercaptopropionylglycine, 8-(p-Sulfophenyl)theophylline and HOE 140 in desflurane treated preparations

Desflurane-induced enhanced recovery of FoC at the end of reoxygenation was abolished (*P *< 0.0001) in presence of MPG (FoC_60_: 54 ± 3% of baseline), SPT (FoC_60_: 62 ± 9% of baseline), HOE 140 (FoC_60_: 58 ± 6% of baseline).

As compared to the control group (control: 51 ± 8% of baseline), MPG alone (MPG: 56 ± 2% of baseline; *P *= 0.08), SPT alone (SPT: 59 ± 3% of baseline; *P *= 0.06), and HOE 140 alone (HOE 140: 54 ± 7% of baseline; *P *= 0.29) did not significantly modify FoC_60 _(fig. 2).

### Effects of adenosine and bradykinin on hypoxia reoxygenation

**Figure 3:**
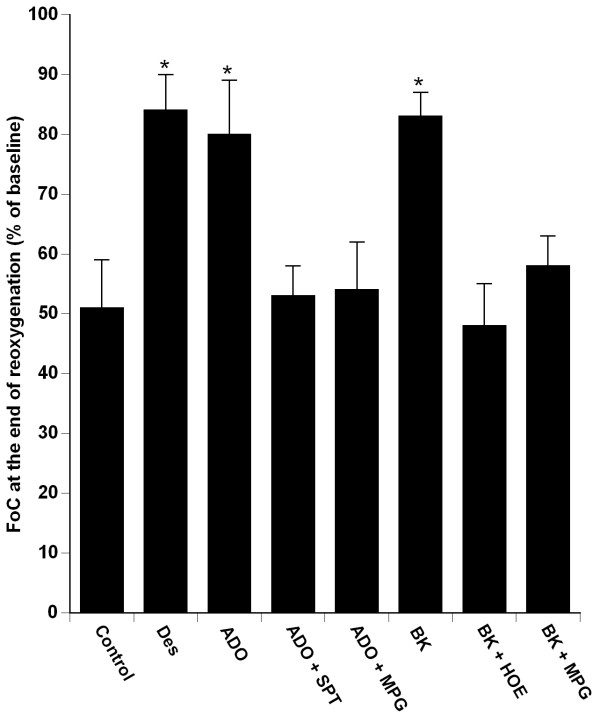
**Recovery of force of contraction of isolated human right atrial trabeculae at the end of the 60-min reoxygenation period after the 30-min hypoxic challenge in groups exposed to Desflurane 6% (Des) alone, exposed to adenosine (ADO) alone or in presence of SPT, MPG; exposed to bradykinin (BK) alone or in presence of HOE, MPG**. Control and Des groups are the same that presented in figure 2. Data are mean ± SD. *P < 0.0001 *vs*. control, ADO + SPT, ADO + MPG, BK + HOE, BK + MPG. ADO: adenosine, BK: bradykinin; Des: desflurane; HOE: HOE 140; MPG: N-mercaptopropionylglycine; SPT: 8-(p-Sulfophenyl) théophylline.

Administration of adenosine and bradykinin, during the first 15 min of the reoxygenation period, significantly increased the recovery of FoC_60 _as compared to the control group (80 ± 9% of baseline in adenosine group and 83 ± 4% of baseline in bradykinin group; *vs*. Control group, *P *< 0.0001). Recovery of FoC_60 _measured in presence of adenosine or bradykinin were similar to that measured in Desflurane group (respectively *P *= 0.37 and *P *= 0.74 *vs*. desflurane group) (fig. 3).

### Effects of 8-(p-Sulfophenyl)theophylline and N-mercaptopropionylglycine on adenosine administration

The effect of adenosine on FoC_60 _was abolished by pretreatment with SPT (FoC_60: _53 ± 8% of baseline), and with MPG (FoC_60: _54 ± 8% of baseline) (*P *< 0.0001 *vs*. adenosine group) (fig. 3).

### Effects of HOE 140 and N-mercaptopropionylglycine on bradykinin administration

The enhanced recovery of FoC_60 _induced by bradykinin was abolished by the pre-treatment with HOE140 (48 ± 8% of baseline), and with MPG (55 ± 3% of baseline) (*P *< 0.0001 *vs*. bradykinin group) (fig. 3).

## Discussion

In the present study, we showed that the cardioprotection triggered by desflurane during early reoxygenation involved ROS generation, and stimulation of adenosine and bradykinin B_2 _receptors. Furthermore, adenosine and bradykinin-induced PostC involved at least in part, ROS production.

The present study shows that administration of MPG abolished desflurane-induced PostC in human myocardium, *in vitro*. These results strongly suggest that desflurane-induced PostC involved ROS production. At present, only two studies have shown, in mouse and rat heart, that isoflurane and sevoflurane-induced PostC was abolished by treatment with ROS scavengers [3,8]. In contrast, numerous studies have shown that ROS play a fundamental role in anesthetic-induced preconditioning [14,16]. Müllenheim *et al*. demonstrated that ROS scavengers, blocked the reduction in myocardial infarct size resulting from isoflurane-induced preconditioning [16]. Isoflurane-induced preconditioning was mediated by ROS generated from electron transport chain complex III, in rabbit heart [17]. Moreover, in ventricular myocytes volatile anesthetics inhibit complex I of the electron transport chain [18], and, ROS mediated the mitochondrial uncoupling induced by desflurane treatment [19]. Tanaka *et al*. reported that mitochondrial adenosine triphosphate-sensitive potassium (mitoK_ATP_) channel opening triggered isoflurane-induced preconditioning via ROS generation, in rabbit *in vivo *[20]. We have previously shown that opening of mitoK_ATP _was an essential step of desflurane-induced PostC in human myocardium [7]. Thus, it could be hypothesized that desflurane-induced PostC generates ROS via mitoK_ATP _channels opening. Further studies are required to examine the exact relationship between mitoK_ATP _channel opening and ROS generation during early reperfusion.

The role of adenosine and bradykinin receptors' stimulation in desflurane-induced PostC was studied here for the first time using human myocardium. The present results show that both adenosine and B_2 _bradykinin receptors' stimulation are involved in desflurane-induced PostC. Pre-treatment with adenosine receptors inhibitor (SPT) and specific B_2 _bradykinin receptor' inhibitor (HOE140) abolished desflurane-induced PostC. Similarly, the beneficial effects of ischemic PostC were abolished by SPT, in rat and rabbit hearts [10,21,22]. Penna et *al*. showed that HOE 140 infusion eliminated the cardioprotective effect induced by ischemic PostC, in isolated rat hearts [11]. Additionally, a growing body of evidence supports the concept that anesthetic PostC triggers a cardioprotective cascade of molecular signalling events similar to that of ischemic PostC [23]. Then, previous studies showed specific involvement of A_2A _and A_3 _adenosine receptors in ischemic PostC in mouse [10,24], and A_1_, A_2B _adenosine receptors in rabbit heart, *in vitro *[25,26]. Using gene knockout mice, it has been shown that ischemic PostC-induced decreased in infarct volume was triggered by activation of adenosine A_1 _and bradykinin B_2 _receptors [27]. In contrast, with anesthetic-induced PostC, the involvement of adenosine receptors (specifically the A_1 _adenosine receptors) stimulation in isoflurane-induced preconditioning has been established [28-30].

Then, we showed that activation of adenosine and bradykinin receptors (via administration of adenosine or bradykinin), at the onset of reoxygenation, enhanced the recovery of FoC_60 _as compared to the control group. Lu *et al*, in rat ventricular myocytes, showed that adenosine-induced PostC was mediated by stimulation of adenosine A_1 _receptors as suggested by abolition of cardioprotective effect of adenosine in presence of 1,3-dipropyl-8-cyclopentylxanthine (DPCPX) as A_1 _adenosine receptor antagonist [31]. Nevertheless, Penna *et al*. showed that a 3-min administration of adenosine at the beginning of reperfusion did not decrease infarct volume in isolated rat hearts [21]. This discrepancy may result from differences in adenosine concentration between studies (i.e. 30 μM in Penna's study *vs *100 μM in Lu's study and our own) and from the brief administration which may be ineffective to stimulate adenosine receptors. Thus, Penna *et al *showed that a 40 min administration of adenosine during reperfusion was necessary to decrease infarct size [21]. In another study, Penna et *al *have shown, in isolated rat hearts, that brief repetitive administration of bradykinin during early reperfusion could trigger PostC, whereas continuous administration in the same period wasn't cardioprotective [11].

Our data showed that pretreatment by HOE 140 abolished bradykinin effects, suggesting that bradykinin effects at the beginning of reoxygenation were mediated by the specific stimulation of B_2 _receptors, in accordance with Penna' s data [11]. Although the exact mechanism by which adenosine and bradykinin protect human myocardium *in vitro *is still unknown, studies based on animal models have suggested that ROS may mediate PostC [11]. Our data show that MPG abolished adenosine- and bradykinin-induced postC, suggesting that adenosine and bradykinin receptors' activation triggered PostC, at least in part, via ROS generation during early reoxygenation. In addition to ROS production, it has been shown that activation of adenosine and bradykinin receptors during reperfusion may promote mitoK_ATP _channels opening [11,31], activation of the RISK pathway including pro survival PI3K/Akt and MEK/ERK 1/2 pathways, p70s6K activation [15,32,33], and phosphorylation of GSK-3β, and prevent the mPTP opening, in rat ventricular myocytes and in isolated rat heart [34,35] These signalling steps have been shown to be involved in ischemic and anesthetic induced PostC. Nevertheless, one study showed that preconditioning triggered by adenosine did not include ROS signaling in isolated rabbit heart [36].

Finally, enhanced recovery of force of human atrial trabeculae after exposure to desflurane, adenosine and bradykinin may suggest an anti-stunning effect of PostC. Whereas PostC reduced infarct size, it does not protect against myocardial stunning in dogs and rabbits [37], moreover post-ischemic systolic function was not modify by ischemic PostC and adenosine as compared to control group in isolated rat heart [21]. Definitely, our results cannot be extrapolated to myocardial stunning which is defined as a completely reversible myocardial dysfunction, because in the present experimental model the contractile dysfunction was not reversible even after 2 hours of reoxygenation [38].

Several limitations must be considered when interpreting our results. First the effects of anesthetic drugs, diseases, or medical treatments received by the patients before obtaining atrial appendages cannot be ruled out. Furthermore, although our experimental groups showed comparable demographic data (table 1), age has been shown to impair ischemic PostC in senescent mouse hearts [39-41], and should also be considered in patients. Second, our experiments were performed under moderate hypothermia (34°C) to ensure stability of trabeculae over time. However, during surgical procedures moderate hypothermia may occur. Third, blockade of adenosine receptors with SPT was not isoform specific, and we have investigated the role of ROS production using MPG. We did not directly measure ROS production nor the particular species of ROS. Fourth, as described in myocardial preconditioning, the beneficial effects of PostC have also been described on reperfusion-induced arrhythmias [42] and myocardial conctractility [43]. Fifth, we measured recovery of post hypoxic contractile function but not the infarct volume. However, it is not possible to precisely quantify the volume of necrosis in isolated trabeculae. Furthermore, it has been shown that the improved recovery of contractile function produced by preconditioning was proportional to reduced infarct size [44]. Finally, previous studies from our laboratories clearly showed that contractile dysfunction is a reliable parameter.

## Conclusion

We have shown that stimulation of adenosine and bradykinin B_2 _receptors and ROS generation, during early reoxygenation, were involved in desflurane-induced PostC in human myocardium, *in vitro*. Additionally, the cardioprotective effect of adenosine and bradykinin administered at the beginning of reoxygenation, was mediated, at least in part, through ROS production.

## Competing interests

The authors declare that they have no competing interests.

This work was supported by the Université de Caen Basse Normandie and Centre Hospitalier Universitaire de Caen.

## Authors' contributions

SL and JLH designed the investigation, reviewed the literature and drafted the manuscript. SL, GB, CI, MM conducted the experiments. JLG, CB and RR contributed to the interpretation of the data.

All authors read and approved the manuscript.

## Pre-publication history

The pre-publication history for this paper can be accessed here:

http://www.biomedcentral.com/1471-2253/10/12/prepub
